# Topic detection using paragraph vectors to support active learning in systematic reviews

**DOI:** 10.1016/j.jbi.2016.06.001

**Published:** 2016-08

**Authors:** Kazuma Hashimoto, Georgios Kontonatsios, Makoto Miwa, Sophia Ananiadou

**Affiliations:** a Graduate School of Engineering, University of Tokyo, Tokyo, Japan; b School of Computer Science, National Centre for Text Mining, University of Manchester, Manchester, United Kingdom; c Department of Advanced Science and Technology, Toyota Technological Institute, Nagoya, Japan

**Keywords:** Systematic reviews, Citation screening, Topic modelling, Paragraph vectors, Document embeddings, Active learning

## Abstract

Systematic reviews require expert reviewers to manually screen thousands of citations in order to identify all relevant articles to the review. Active learning text classification is a supervised machine learning approach that has been shown to significantly reduce the manual annotation workload by semi-automating the citation screening process of systematic reviews. In this paper, we present a new topic detection method that induces an informative representation of studies, to improve the performance of the underlying active learner. Our proposed topic detection method uses a neural network-based vector space model to capture semantic similarities between documents. We firstly represent documents within the vector space, and cluster the documents into a predefined number of clusters. The centroids of the clusters are treated as latent topics. We then represent each document as a mixture of latent topics. For evaluation purposes, we employ the active learning strategy using both our novel topic detection method and a baseline topic model (i.e., Latent Dirichlet Allocation). Results obtained demonstrate that our method is able to achieve a high sensitivity of eligible studies and a significantly reduced manual annotation cost when compared to the baseline method. This observation is consistent across two clinical and three public health reviews. The tool introduced in this work is available from https://nactem.ac.uk/pvtopic/.

## Highlights

• We propose a topic detection method based on paragraph vectors.
• The method is integrated with an active learner to accelerate citation screening.
• The method outperforms LDA when applied to clinical and public health reviews.

## Graphical abstract

**Figure:**
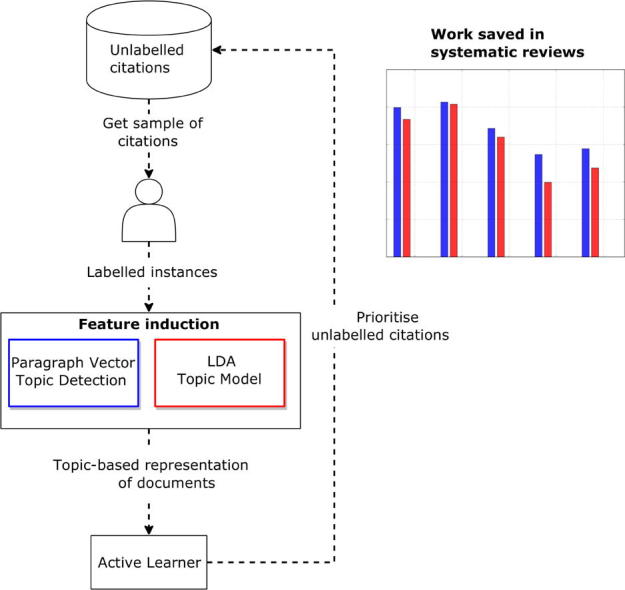

## 1 Introduction

Systematic reviews involve searching, screening and synthesising research evidence from multiple sources, in order to inform policy studies and guideline development [1]. In evidence-based medicine, systematic reviews are vital in guiding and informing clinical decisions, and in developing clinical and public health guidance [2]. In carrying out systematic reviews, it is critical to minimise potential bias by identifying all studies relevant to the review. This requires reviewers to exhaustively and systematically screen articles for pertinent research evidence, which can be extremely time-consuming and resource intensive [3].

To reduce the time and cost needed to complete the screening phase of a systematic review, researchers have explored the use of active learning text classification to semi-automatically exclude irrelevant studies while keeping a high proportion of eligible studies (i.e., sensitivity of at least 95%) in the final review [4], [5], [6]. Active learning text classification is an iterative process that incrementally learns to discriminate eligible from ineligible studies. The process starts with a small seed of manually labelled citations that is used to train an initial text classification model. The active learner will then iterate through several learning cycles to optimise its prediction accuracy. At each learning cycle, the active learner automatically classifies the remaining unlabelled citations. A sample of the automatically labelled citations is validated by an expert reviewer. Finally, the validated sample is used to update (re-train) the classification model. The process terminates when a convergence criterion is satisfied (e.g., 95% of eligible studies is identified by the active learner).

Key to the success of the active learning approach is the feature extraction method that encodes documents into a vector representation that is subsequently used to train the text classification model. Wallace et al. [5] proposed a multi-view active learning approach that represents documents using different feature spaces, e.g., words that appear in the title and in the abstract, keywords and MeSH terms. Each distinct feature space is used to train a sub-classifier, e.g. Support Vector Machines (SVM). Multiple sub-classifiers are then combined into an ensemble classifier using a heuristic (e.g., majority votes). With regard to the active learning selection criterion (i.e., a function that determines the next sample of instances to be validated by the reviewer), the authors employed uncertainty sampling. The uncertainty selection criterion selects those instances for which the classifier is least certain of their classification label. To enhance the performance of the active learner, they introduced an aggressive undersampling technique that removes ineligible studies from the training set which convey little information. The aggressive undersampling technique aims at reducing the negative effect of class imbalance that occurs in systematic reviews, i.e., a high percentage of ineligible studies tends to overwhelm the training process. For experimentation, they applied the proposed method to three clinical systematic review datasets. They showed that the uncertainty-based active learner with aggressive undersampling is able to decrease the human-workload involved in the screening phase of a systematic review by 40–50%.

Whilst good results are obtained in the clinical domain, Miwa et al. [4] demonstrated that the active learning approach yields a significantly lower performance when applied to public health reviews. The authors argued that the identification of relevant studies is more challenging in this domain compared to others, e.g., clinical documents. This can be attributed to the fact that the public health literature extends across a wide range of disciplines covering diverse topics (e.g., social science, occupational health, education, etc.) [7]. To alleviate problems introduced by challenging public health articles, the authors proposed to learn a topic-based representation of studies by employing the widely used Latent Dirichlet Allocation (LDA) [8], a probabilistic and fully generative topic model. They further investigated the use of a certainty-based selection criterion that determines a validation sample consisting of instances with a high probability of being relevant to the review (as opposed to the previously introduced uncertainty sampling [5] that selects instances with low classification probability). Experimental results determined that topic-based features can improve the performance of the active learner. Moreover, the certainty-based active learner that uses topic features induced by LDA exceeded state-of-the-art performance and outperformed the uncertainty-based active learner [5].

Topic models are machine learning methods that aim to uncover thematic structures hidden in text. One of the earliest topic modelling methods is the probabilistic Latent Semantic Indexing (PLSI) [9]. PLSI associates a set of latent topics *Z* with a set of documents *D* and a set of words *W* (*D*, *W* are observed variables). The goal is to determine those latent topics that best describe the observed data. In PLSI the probability distribution of latent topics is estimated independently for each document. In practice, this means that the complexity of the model (i.e., number of parameters to be computed) grows linearly with the size of the collection. A further disadvantage of PLSI is the inability of the underlying model to generalise on new, unseen documents (i.e. the model is not fully generative). Extending upon of PLSI, LDA assumes that topic distributions are drawn from the same prior distribution which allows the model to scale up to large datasets and better generalise to unseen documents.

In this article, we present a novel topic detection model to accelerate the performance of the active learning text classification model used for citation screening. Our topic detection method can be used as an alternative approach to the LDA topic model to generate a topic-based feature representation of documents. The proposed method uses a neural network model, i.e., paragraph vectors [10], to learn a low dimensional, but informative, vector representation of both words and documents, which allows detection of semantic similarities between them. Previous work has demonstrated that paragraph vector models can accurately compute semantic relatedness between textual units of varying lengths, i.e., words, phrases [11] and longer sequences, e.g., sentences, paragraphs and documents [10]. While the standard bag-of-words approach (i.e., a document is represented as a vector of the words that it contains) has been frequently employed in various natural language processing tasks (e.g., text classification, sentiment analysis), paragraph vectors, which take into account factors such as word ordering within text, have been shown to yield superior performance [10].

To our knowledge, our work is the first that utilises the vector representations of documents produced by the paragraph vector model for topic detection. We hypothesise that documents lying close to each other in the vector space form topically coherent clusters. Based on this, our approach clusters the paragraph vector representations of documents by applying the *k*-means clustering algorithm and treats the centroids of the clusters as representatives of latent topics, assuming that each cluster corresponds to a latent topic inherent in the texts. After detecting latent topics in a collection of documents, we represent each document as a *k*-dimensional feature vector by calculating the distance of the document to the *k* cluster centroids. Additionally, our topic detection model computes the conditional probability that a word is generated by a given topic and thus readily determines a set of representative keywords to describe each topic. The topic-based representation of documents is used to train an active learning text classification model to more efficiently identify eligible studies for inclusion in a review. The contributions that we make in this paper can be summarised in the following points:

1. We propose a novel topic detection method that builds upon the paragraph vector model. We introduce various adaptations to the paragraph vector method that enable the underlying model to discover latent topics in a collection of documents and summarise the content of each topic by meaningful and comprehensive text labels.
2. We incorporate the new topic detection method with an active learning strategy to support the screening process of systematic reviews.
3. We conduct experiments, demonstrating that our topic detection method outperforms an existing topic modelling approach when applied to semi-automatic citation screening of clinical and public health reviews.

## 2 Methods

In this section, we detail our proposed topic detection method. We then provide an overview of the active learning process used in our experiments and discuss the evaluation protocol that we follow to asses the paragraph vector-based topic detection method.

### 2.1 A paragraph vector-based topic detection method

#### 2.1.1 Word vectors

Several approaches on representing the meaning of words using mathematical expressions such as vectors and matrices have been proposed, with neural network models recently gaining much attention [11], [12], [13]. Neural network models usually fully parameterise the word vectors; in other words, each word *w* has *n* parameters in its word vector: $v(w)=(x_{w1}\text{,}x_{w2}\text{,}\ldots \text{,}x_{\mathrm{wn}})$. The parameters are used to estimate the conditional probability that a target words will appear, given its context words. The parameters for each word are initialised with random values, and then adjusted in the learning process whose objective is to maximise the conditional probability:

(1) $$p(w_{t}|w_{t-N}\text{,}w_{t-N+1}\text{,}\ldots \text{,}w_{t-1})$$

where $w_{t}$ is the target word and $w_{t-N}\text{,}w_{t-N+1}\text{,}\ldots \text{,}w_{t-1}$ are *N* context words that occur before $w_{t}$. During the learning process, the parameterised vectors of the context words are used and updated, and the vectors of words which appear in similar contexts (i.e., used in similar contexts) are updated similarly. As a result, the vectors of words which are likely to appear in similar contexts appear close to each other in the vector space. Such word vectors have proven to be useful in many NLP tasks, e.g., part-of-speech tagging and named entity recognition [13], [14]. The trained models can then be used to predict the target word with the highest conditional probability, given its context words.

#### 2.1.2 Paragraph vectors

More recently, a neural network model has been proposed that is able to induce word vectors and paragraph vectors jointly [10]. While word vectors represent only words, paragraph vectors represent phrases, sentences, paragraphs and documents of arbitrary length. In this work, we use paragraph vectors to model documents. Given a document *d*, its representation is defined as a parameterised vector v(d), in the same way as for word vectors. In this model, the vector parameters are adjusted simultaneously to predict target words according to their context words and documents in a similar way to the previously described word vector learning method [11]. The probability that a target word will appear in a given context is conditioned not only by the context words but also by the document:

(2) $$p(w_{t}|w_{t-N}\text{,}w_{t-N+1}\text{,}\ldots \text{,}w_{t-1}\text{,}d)=\sigma (s(w_{t})\cdot [v(w_{t-N})\text{;}v(w_{t-N+1})\text{;}\ldots \text{;}v(w_{t-1})\text{;}v(d)])$$

where $s(w_{t})$ is a weight vector for computing the conditional probability, σ(·) is the logistic function, [·;·] is the concatenation of vectors, and *d* denotes the document including the sequence of words. By modelling and maximising the probability using both word and paragraph vectors, the paragraph vectors are adjusted to capture co-occurrence statistics of words within the documents. Just as word vectors capture similarities between words, paragraph vectors capture similarities between documents. Paragraph vectors representing documents covering the same topic are thus likely to lie close to each other in the vector space. Recent work has used paragraph vectors to detect similarities between Wikipedia articles and research papers [15].

#### 2.1.3 Topic detection by clustering paragraph vectors

Topic models assume that a set of documents has a specific number of latent topics, and words in a document are probabilistically generated, given the document’s topics. For example, if a topic assigns high probabilities to the words “alcohol”, “drunk”, and “accidents”, we can infer that the topic is about alcohol-related accidents. Our novel contribution is the development of a topic detection method using the paragraph vector model. To aid the study identification process of systematic reviews, it is useful to capture semantic similarities between articles and group studies according to the latent topics within them. Since typical approaches to topic models are based on bags-of-words, important information that can be used to calculate semantic similarity, e.g., word order, is lost [16], [17]. In contrast, the paragraph vectors approach allows us to incorporate more detailed contextual information into our topic detection method.

**Fig. 1:**
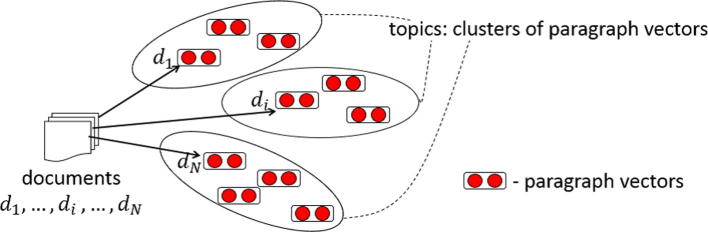
Detecting latent topics using paragraph vectors.

Fig. 1 shows an overview of our proposed topic detection method. To detect the latent topics inherent in a set of documents, we first cluster the learned paragraph vectors using the *k*-means clustering algorithm to obtain *K* cluster centres of the paragraph vectors. As a distance metric for *k*-means clustering, we use the cosine similarity between paragraph vectors (i.e., spherical *k*-means [18]). Whilst alternative distance metrics could be used in *k*-means clustering (e.g., Euclidean distance), previous work has demonstrated that the cosine of the angle between word or paragraph vectors provides robust results [11], [15]. We treat the *K* cluster centre vectors $v(c_{1})\text{,}v(c_{2})\text{,}\ldots \text{,}v(c_{K})$ as the representations of the *K* latent topics $\left(c_{1}\text{,}c_{2}\text{,}\ldots \text{,}c_{K}\right)$. We then derive a *K*-dimensional topic-based representation $\left(t_{1}\text{,}t_{2}\text{,}\ldots \text{,}t_{i}\text{,}\ldots \text{,}t_{k}\right)$ of a document by considering the dot product between the paragraph vector v(d) of the document and the paragraph vectors $v(c_{1})\text{,}v(c_{2})\text{,}\ldots \text{,}v(c_{K})$ of the *K* cluster centroids. The *i*-th feature value of the topic-based document vector determines the degree of correlation between the document and the *i*-th latent topic and is calculated as follows:

(3) $$t_{i}=\frac{\exp(v(d)\cdot v(c_{i}))}{\sum_{j}\;\exp(v(d)\cdot v(c_{j})}$$

#### 2.1.4 Inducing topic descriptors

Given that paragraph vectors are trained by solving word prediction tasks, we can compute the conditional probability $p(w|d_{w})$ that a word *w* is generated given a document $d_{w}$. Concretely, the probability is computed by omitting the context information in Eq. (2):

(4) $$p(w_{t}|p_{d_{w}})$$

Using the cluster centre vectors, we can determine a set of words that best describe a given topic, since the cluster centre vectors are in the same vector space as the paragraph vectors. The probability $p(w|c_{i})$ that a word *w* is generated given the *i*-th (1⩽i⩽K) topic is computed by normalising the prediction scores for the words given the *i*-th topic as follows:

(5) $$p(w|c_{i})=\frac{\exp(s(w)\cdot v(c_{i}))}{\sum_{j}\;\exp(s(w_{j})\cdot v(c_{i}))}$$

where $s(w_{j})$ is the weight vectors for calculating the word prediction scores in the paragraph vector model.

#### 2.1.5 An example of topics and descriptive topic labels

**Fig. 2:**
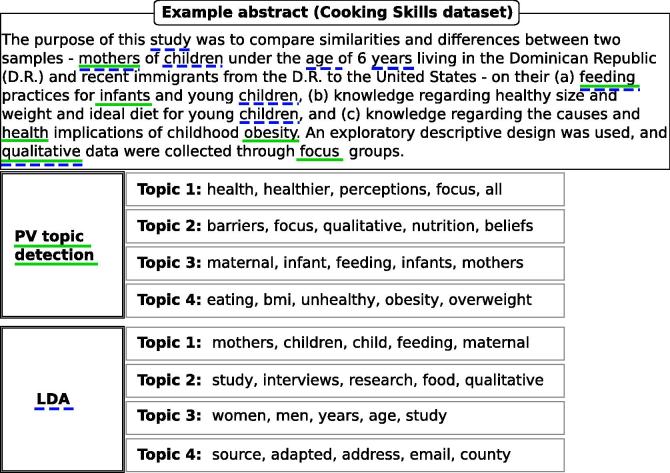
Examples of topics and descriptive topic labels extracted by the paragraph vector-based topic detection method (i.e., PV topic detection) and the LDA topic model from an abstract within the Cooking Skills dataset. Topic labels that are present in the abstract are highlighted with solid green lines for the paragraph vector-based topic detection method and with dashed blue lines for LDA. (For interpretation of the references to colour in this figure legend, the reader is referred to the web version of this article.)

Fig. 2 shows an example abstract from the Cooking Skills dataset (Table 1 summarises the characteristics of the dataset) with the 4 most important topics induced by the proposed topic detection (i.e., PV topic detection) method and the LDA topic model. The two topic detection methods are trained by setting the number of topics to 300. Moreover, each topic is characterised by the top 5 words (i.e., descriptive topic labels) with the highest probability of being relevant to that topic. An exact match between words that occur in the abstract and the topic descriptors is highlighted by a solid green line for the PV topic detection method and with a dashed blue line for the LDA topic model.

The automatically assigned topic descriptions show that the two topic detection methods tend to induce thematically coherent topics which are also representatives of the underlying abstract. For example, topics 3 and 4 extracted by the paragraph vector-based topic detection method seem to be related to two of the key points discussed in the abstract (i.e., *“…* *feeding practices for infants and young children…”* and “*childhood obesity*”). Moreover, it can be noted that both models capture synonymous or semantically related words that occur as keywords in the same topic (e.g., ‘mother/maternal’, ‘overweight/obesity’).

### 2.2 Evaluation settings

#### 2.2.1 Evaluation method

**Fig. 3:**
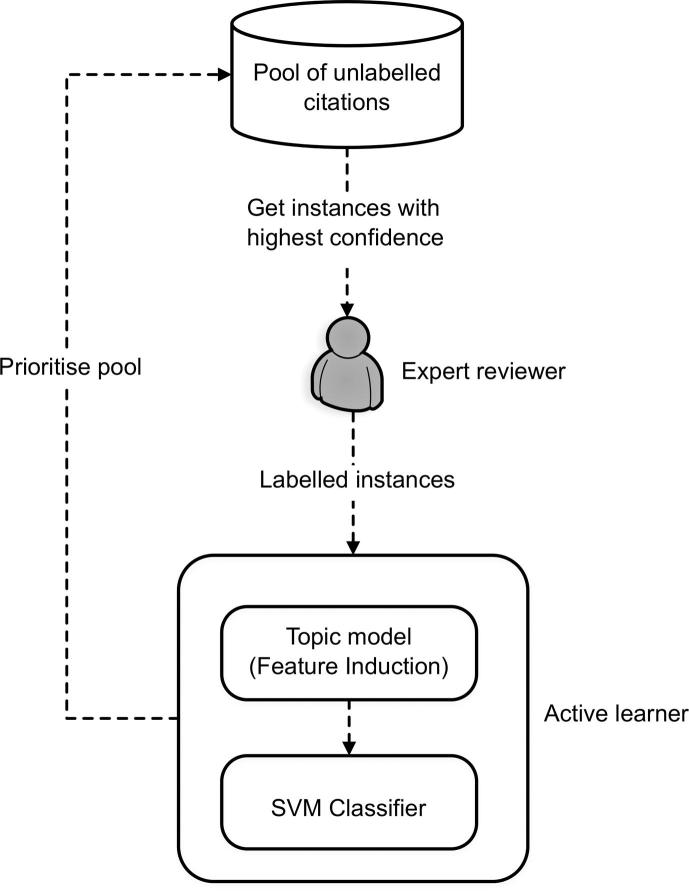
High-level view of a certainty-based active learning strategy [4] used for citation screening.

To evaluate the proposed topic detection method, we investigate the performance of a certainty-based active learning classifier using topic-based features extracted by our paragraph vector-based method and the baseline LDA model. We employ a certainty-based active learning classifier, previously presented in Miwa et al. [4]. A high-level view of the active learning strategy is illustrated in Fig. 3. In our approach, citations are represented as a mixture of topics induced by a topic modelling approach (e.g., the proposed topic detection method or LDA). The two topic models used in this work are unsupervised methods. Thus, we extract topics from the complete set of citations.

An expert reviewer initiates the active learning process by manually labelling a small sample of citations. This labelled sample, encoded into a topic-based representation, is then used to train an SVM text classification model. The trained model automatically classifies the remaining unlabelled citations and determines the next sample of citations to be validated by the reviewer according to a certainty-based criterion, i.e., instances for which the classifier has assigned a high confidence value of being relevant to the review. The certainty selection criterion has been previously shown to better address class imbalance (i.e., a significantly skewed distribution of eligible and ineligible studies) [4], [19] that occurs in systematic reviews. In a succeeding iteration, the reviewer validates the next sample of citations which is used to augment the training set with additional labelled instances. The iterative process terminates when at least 95% of eligible studies are identified by the active learner [3], [4], [5], [6], ideally without needing to manually label the entire list of citations.

In our experiments, we simulate a human feedback active learning strategy [4], [5] given that the employed datasets are already manually coded with gold standard classification labels. At each learning iteration, we construct a sample of 25 studies (i.e., instances for which the classifier has assigned the highest probability of being relevant to the review) and we validate the sample against the gold standard. The validation sample is subsequently used to re-train the text classification model. Following previous approaches, we repeat learning iterations until the active learner has screened the complete list of citations.

### 2.3 Datasets

We report the performance of the active learner when applied to the first stage of the screening process (i.e., screening of titles and abstracts). Cross-validation experiments are performed on two publicly available clinical datasets [5] and three public health datasets, previously used in Miwa et al. [4]. Table 1 summarises the five datasets that we use for experimentation accompanied with the: (a) underlying domain, (b) number of citations and (c) percentage of eligible studies. It is noted that the size of the five employed datasets varies significantly, from small clinical review of approximately 1600 citations (i.e., COPD) to a large public health review of more than 15,000 citations (i.e., Youth Development). Additionally, all five datasets contain a very low percentage of eligible studies that range between 2% and 12%.

#### 2.3.1 Settings for machine learning methods

In order to maximise the performance of the active learner, we tune the parameters of the topic modelling methods. Specifically, we train the paragraph vector-based topic detection method by setting the dimensionality of word vectors to 300, the dimensionality of document vectors to 1000 and the number of training epochs to 500. We then applied the *k*-means algorithm to cluster the paragraph vectors into 300 clusters which resulted in a topic-based representation of 300 dimensions. With regard to the baseline LDA topic model, we used the freely available MALLET toolkit [20]. Additionally, we performed hyperparameter optimisation for every 10 Gibbs sampling iterations and set the total number of iterations to 500. As in the case of the proposed topic detection method, we used 300 LDA topics to represent documents. To train an SVM text classification model, we used the LIBLINEAR library [21] with a dual L2-regularised L2-loss support vector classification solver.

#### 2.3.2 Evaluation metrics

We evaluate the performance of the active learning process, over different learning iterations, using two metrics, namely Yield and Burden [4], [5]. Yield determines the percentage of eligible studies identified by the active learner while burden the percentage of studies that are manually labelled (i.e., manual annotation cost). The overall goal of active learning is to achieve a high yield performance of at least 95% [3], [4], [6] while minimising the screening burden. We calculate yield and burden as follows:

(6) $$\mathrm{yield}=\frac{{\mathrm{TP}}^{M}+{\mathrm{TP}}^{A}}{{\mathrm{TP}}^{M}+{\mathrm{TP}}^{A}+{\mathrm{FN}}^{A}}$$

(7) $$\mathrm{burden}=\frac{{\mathrm{TP}}^{M}+{\mathrm{TN}}^{M}+{\mathrm{TP}}^{A}+{\mathrm{FP}}^{A}}{N}$$

where *N* is the total number of citations, TP,TN,FP and *FN* the number of true positive (eligible studies), true negative (ineligible studies), false positive (studies that are incorrectly classified as eligible) and false negative instances (studies that are incorrectly classified as ineligible), where the superscript *M* and *A* denote manual and automatic screening decisions, respectively. In the definition of burden, the sum ${\mathrm{TP}}^{M}+{\mathrm{TN}}^{M}$ represents the number of studies that are manually labelled and used for training the system while ${\mathrm{TP}}^{A}+{\mathrm{FP}}^{A}$ is the number of studies that are automatically classified as being relevant to the review but still need to be manually validated by a human reviewer in order to be included in the final review.

As a further evaluation, we use the work saved over sampling at 95% recall (WSS@95%) which shows the percentage of ineligible citations that can be safely and automatically excluded (i.e., reviewers do not need to manually validate these instances for inclusion in the review) when the underlying active learner yields a recall performance of 95%. Previous approaches [6] used the WSS@95% metric to evaluate the performance of automatic classification approaches that takes into consideration only automatic screening decisions. In an active learning scenario, WSS@95% can be estimated as follows:

(8) WSS@95%=(1-burden)over a yield performance of95%

## 3 Results

We investigate the performance of active learning, in terms of yield and burden, over an increasing number of manually labelled instances that are used for training. During the last iteration of the active learning process, both yield and burden are 100% since the active learner has identified all eligible studies but with the maximum manual annotation cost (i.e., the complete citation list is manually screened).

**Fig. 4:**
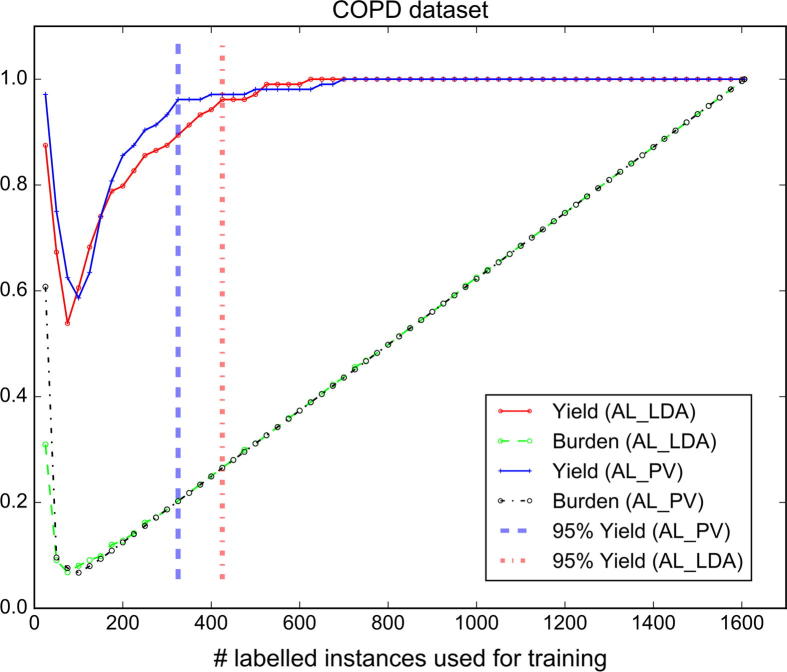
Performance (yield and burden) achieved by the AL_LDA and AL_PV models when applied to the clinical COPD dataset.

**Fig. 5:**
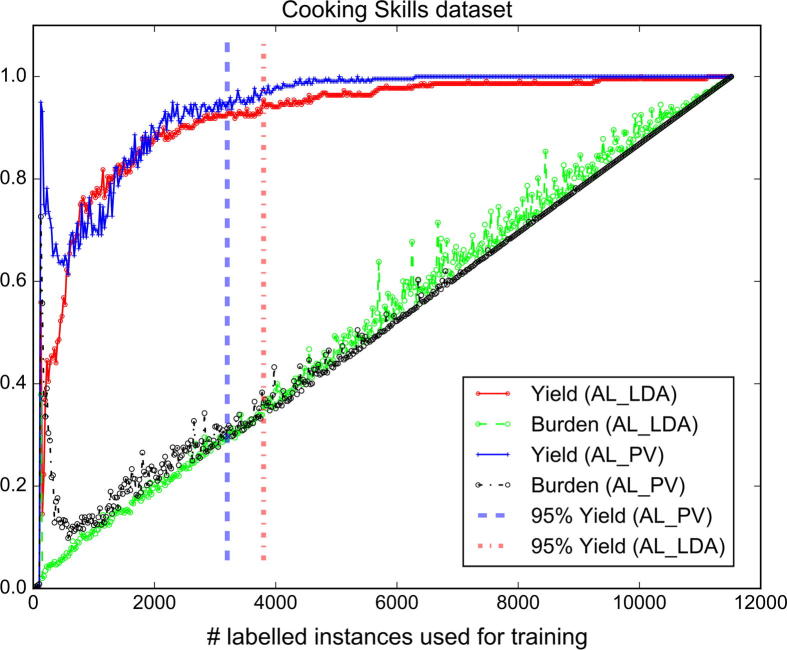
Performance (yield and burden) achieved by the AL_LDA and AL_PV models when applied to the public health Cooking Skills dataset.

Fig. 4, Fig. 5 show the yield and burden performance achieved by the active learning models when applied to the COPD and Cooking Skills datasets, respectively (please refer to the supplementary material for the yield and burden performance of the models on the other datasets). We denote with AL_PV an active learning model that uses topic features extracted by our proposed paragraph vector-based topic detection method and with AL_LDA the baseline active learning model that employs LDA topic features. The dashed vertical lines indicate when an optimal yield performance of 95% is reached. In all cases, the burden performance follows a U-shaped pattern. This can be explained by the fact that during the initial learning iterations where a small number of instances is available for training, the active learner erroneously predicts that the majority of studies is relevant to the review which results in an increased screening burden. As we extend the training set with more labelled instances, the burden performance descends since the active learner obtains a more stable classification performance. Finally, the screening burden increases again but this time linearly (although some fluctuations are observed) with the number of labelled instances.

In the clinical COPD dataset, the AL_PV method shows approximately the same burden performance with the AL_LDA model. However, our active learning strategy converged faster to a high yield value when compared to the baseline AL_LDA method. The AL_PV method improved the yield performance of the baseline model by approximately 3–7% in the COPD dataset. For a given manual annotation workload of 17% (i.e., 17% of the instances were manually labelled), the AL_PV method automatically identified 91% of relevant studies compared to 87% of relevant instances retrieved by the AL_LDA method. By increasing the manual annotation workload to 20%, the AL_PV method achieved a yield performance of 96% while the baseline AL_LDA a yield performance of 89%. With regard to the Cooking Skills dataset (i.e., public health review), we observe that during the early learning iterations the performance obtained by the AL_PV model slightly fluctuated and in some cases the model obtained a lower yield and burden performance than the AL_LDA. In subsequent learning iterations, the AL_PV achieved a superior yield and burden performance compared to the baseline.

### 3.1 Reduction of manual annotation workload

In this section, we evaluate the paragraph vector-based topic detection method by computing the work saved over sampling at 95% recall (WSS@95%). We further implement two additional baseline methods, namely the AL_BoW_K-Means and the AL_Average_WV. The two baseline methods follow a similar approach to our proposed method to compute a vector representation of documents. Firstly, they apply the *k*-means algorithm to generate k=300 document clusters. Secondly, they induce a feature representation of documents by computing the distance of a document to the *k* cluster centroids. The AL_BoW_K-Means method uses *k*-means over a standard BoW representation of documents. The AL_Average_WV performs *k*-means clustering over the *mean word representation* of documents, i.e., the average of vectors for words that appear in a document. Word vectors are obtained by the word2vec tool [11] using the same parameter settings as in the paragraph vector model (i.e., 500 training epochs and 300 dimensions for the word vectors). A key difference between the AL_PV and the AL_Average_WV method is that the former approach trains word and document vectors jointly while the latter method trains word vectors alone.

**Fig. 6:**
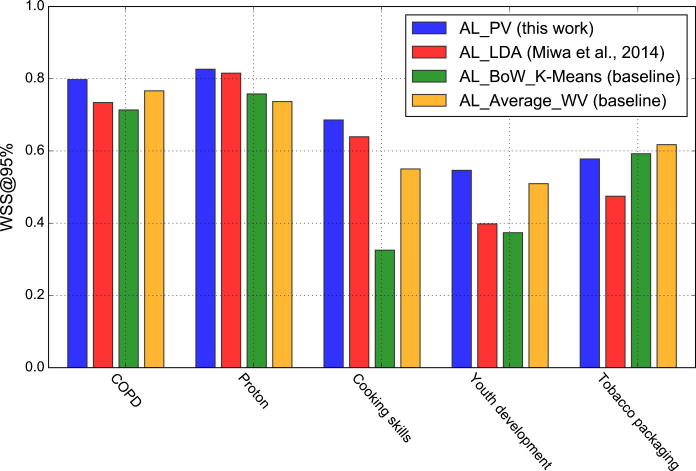
WSS@95% achieved by the AL_PV and AL_LDA active learning models across clinical and public health reviews.

Fig. 6 summarises WSS@95% scores obtained by the four active learning models (AL_PV, AL_LDA, AL_BoW_K-Means and AL_Average_WV) across clinical and public health reviews. It can be noted that in 4 datasets, our proposed active learning method outperformed the AL_LDA by a statistically significant margin.^2^ The improvements varied between 5% in the COPD and Cooking Skills reviews to 10% in the Tobacco packaging and 15% in the Youth development review. For the ProtonBeam review, we observed an insignificant improvement of 1% achieved by AL_PV in comparison to the AL_LDA model. The AL_BoW_K-Means and AL_Average_WV methods obtained a slightly higher WSS@95% performance than the AL_PV model in the Tobacco Packaging review (i.e., 1–4%). However, the AL_PV method surpassed the performance of the two baseline methods in the remaining 4 datasets (i.e., 3–6% in the COPD review, 1–9% in the Proton review, 13–33% in the Cooking Skills review and 4–17% in the Youth Development review).

## 4 Discussion

The experiments that we conducted demonstrate that the proposed topic detection method can improve upon a state-of-the-art semi-automatic citation screening method [4] that employs the standard LDA topic model. In clinical reviews, our topic detection method outperformed the LDA-based model by 1–5% while in public health reviews we observed larger performance gains between 5% and 15% in terms of WSS@95. These results suggest that the paragraph vector-based topic detection model can substantial reduce the manual annotation workload involved in both clinical and public health systematic reviews.

In our approach, we followed a retrospective evaluation protocol [5], [4] where automatic screening predictions were compared against completed systematic reviews. This retrospective evaluation assumes that human reviewers screen at a constant rate which is not always the case in live systematic reviews. For example, O’Mara-Eves et al. [3] outlined that reviewers tend to make faster screening decisions once they have processed the majority of the important studies. Based upon this, we plan to integrate our topic detection method with bespoke systematic review systems [22], [23] and assess the performance of active learning in real application scenarios.

Moreover, we will investigate alternative uses of topic modelling techniques that can further facilitate the study identification phase in systematic reviews. Specifically, although the literature of some disciplines is indexed using well-organised (e.g., using controlled vocabularies) bibliographic databases, e.g., MEDLINE [24] or EMBASE [25], this is not so for all disciplines, which can result in decreased performance of search strategies. Additionally, the PICO framework (Is this intervention (I) effective (Outcome) for this population (P) compared with this other intervention (C)) which is commonly used to structure pre-defined questions matching clinical needs, ill suits public health reviews [26]. Unlike clinical questions, public health questions are complex and may be described using abstract, fuzzy terminology, excluding defining a priori an adequate PICO question. Thus, topic modelling approaches that automatically discover groups of semantically related words and documents can be used to organise the most relevant evidence in a dynamic, interactive way that supports how public health reviews are conducted.

## 5 Conclusions

In this paper, we presented a new topic detection method to support the screening phase of systematic reviews. Our proposed method uses a neural network model to identify clusters of semantically related documents. By treating the cluster centroids as representatives of latent topics, we enable the model to learn an informative and discriminative feature representation of studies. This new topic-based representation of studies is utilised by an active learning text classification model to semi-automatically identify citations for inclusion in a review and thus directly reduce the human workload involved in the screening phase.

We evaluated our approach against an active learning strategy that employs topic-based features extracted by Latent Dirichlet Allocation (LDA) in both clinical and public health reviews. Experimental evidence showed that the neural network-based topic detection method obtained an improved yield and burden performance when compared to the baseline method. Additionally, we demonstrated that in four out of five reviews, the proposed method drastically reduced the manual annotation cost while retaining 95% of eligible studies in the final review.

## Conflicts of interest statement

The authors declare that they have no conflict of interest.

## Figures and Tables

**Table 1:** Characteristics of clinical and social science reviews used for experimentation.

Dataset	Scientific	# citations domain	Ratio of eligible to ineligible studies (%)
COPD	Clinical	1606	12
ProtonBeam	Clinical	4751	5
Cooking Skills	Public health	11,515	2
Tobacco packaging	Public health	3210	5
Youth development	Public health	15,544	10

## Footnotes

2 We used the unpaired *t*-test at p<0.05 to assess statistical significance.
