# Supplementary material for: Topic detection using paragraph vectors to support active learning in systematic reviews
Source: J Biomed Inform. 2016 Aug;62:59–65. doi: 10.1016/j.jbi.2016.06.001 (PMC4981645; doi:10.1016/j.jbi.2016.06.001)
Supplement: Supplementary Figs. 1–5 [file mmc1.pdf]

Supplementary material of the manuscript: "Topic Detection Using Paragraph Vectors to Support the Screening Phase of Systematic Reviews"

Kazuma Hashimoto[1], Georgios Kontonatsios[1], Makoto Miwa[1], Sophia Ananiadou[1,]

[a] *Graduate School of Engineering, University of Tokyo, Tokyo, Japan*
[b] *School of Computer Science, National Centre for Text Mining, University of Manchester, Manchester, United Kingdom*
[c] *Department of Advanced Science and Technology, Toyota Technological Institute, Nagoya, Japan*

1. Introduction

This document provides evaluation results obtained by an active learning strategy that employs both the proposed neural network-based topic detection method and the baseline LDA model. The active learner is applied to two clinical and three public health reviews. The figures below show yield and burden performance achieved by the active learner. Additionally, the dashed vertical lines indicate when an optimal yield performance of 95% is reached. Evaluation results on the COPD and Cooking Skills datasets are also present in the main manuscript.

* corresponding author

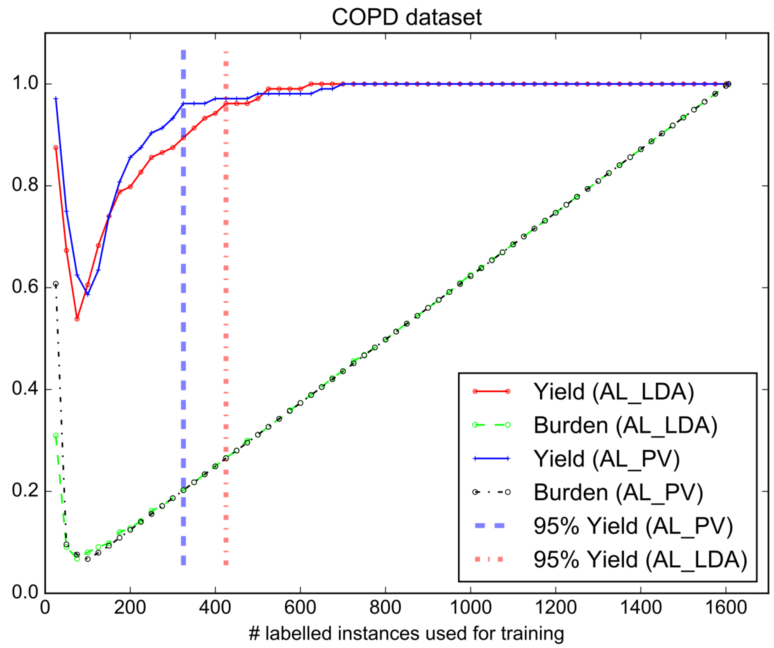

Figure 1: Performance (yield and burden) achieved by the AL_LDA and AL_PV models when applied to the clinical COPD dataset

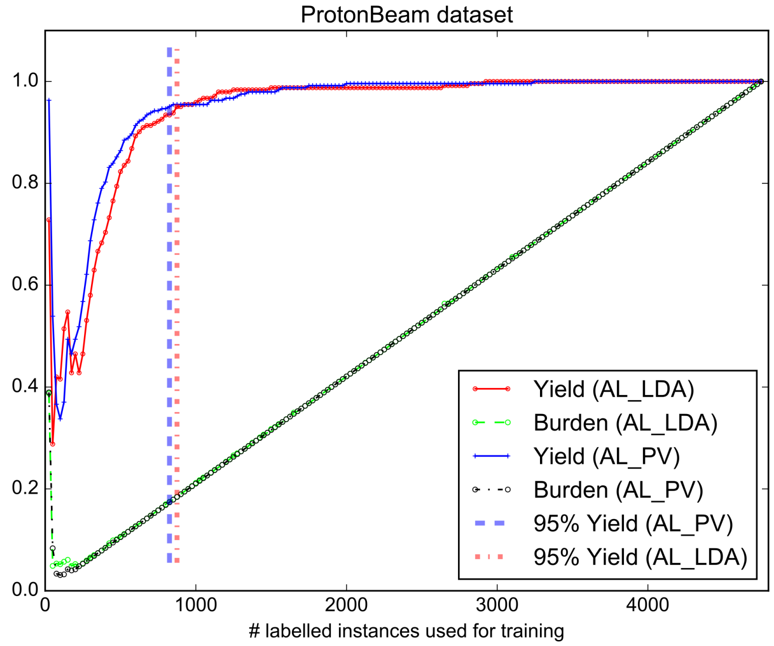

Figure 2: Performance (yield and burden) achieved by the AL_LDA and AL_PV models when applied to the clinical ProtonBeam dataset

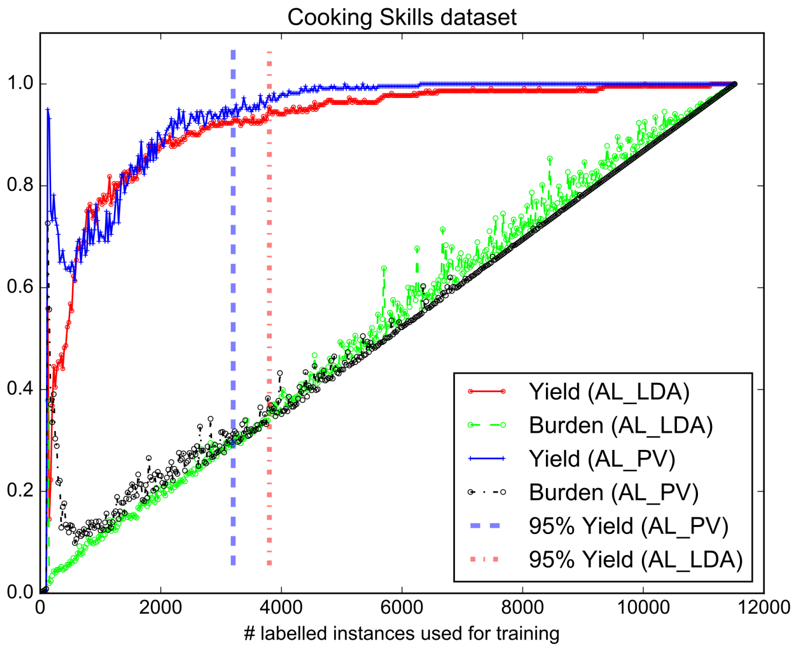

Figure 3: Performance (yield and burden) achieved by the AL_LDA and AL_PV models when applied to the public health Cooking Skills dataset

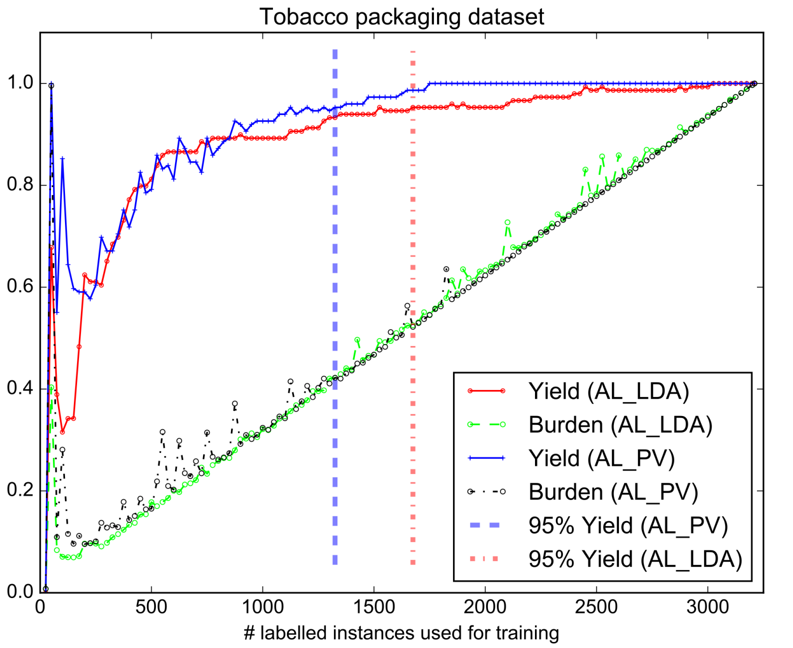

Figure 4: Performance (yield and burden) achieved by the AL_LDA and AL_PV models when applied to the public health Tobacco Packaging dataset

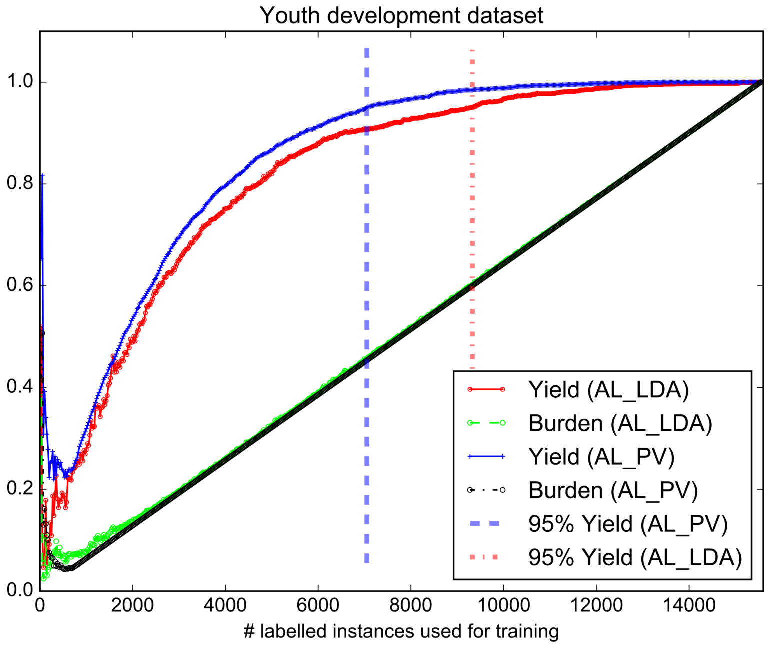

Figure 5: Performance (yield and burden) achieved by the AL_LDA and AL_PV models when applied to the public health Youth Development dataset
